# Hamman's Syndrome: A Rare Cause of Chest Pain in a Postpartum Patient

**DOI:** 10.1155/2015/201051

**Published:** 2015-01-21

**Authors:** Daniyeh Khurram, Brijesh Patel, M. Waseem Farra

**Affiliations:** ^1^Department of Internal Medicine, Providence Hospital and Medical Center, 16001 W Nile Mile Road, Southfield, MI 48075, USA; ^2^Department of Pulmonology, Providence Hospital and Medical Center, 16001 W Nile Mile Road, Southfield, MI 48075, USA

## Abstract

Hamman's syndrome is a rare condition represented by spontaneous pneumomediastinum and subcutaneous emphysema. Excessive Valsalva maneuver during vaginal delivery and excessive retching, coughing, and straining are frequently reported causes. The incidence of Hamman's syndrome is believed to be 1 in 100,000 deliveries. The pathophysiology of this condition is rupture of alveoli and seepage of air through bronchovascular connective tissue. Diffusion of air to subcutaneous tissues results in subcutaneous emphysema. In most cases, it is a benign condition and resolves spontaneously. In life-threatening cases, a cardiac tamponade can ensue. Chest X-ray is a useful early diagnostic technique. We report a case of a twenty-four-year-old female who was diagnosed with Hamman's syndrome after prolonged, exhaustive labor.

## 1. Introduction

Spontaneous pneumomediastinum is defined as the presence of free air in the mediastinum in the absence of an obvious precipitating cause. When associated with subcutaneous emphysema, it is called “Hamman's syndrome” and was initially described by Hamman in a postpartum female who developed pneumomediastinum with subcutaneous emphysema [1]. It may be associated with a crunching sound synchronous with the heartbeat, referred to as the “Hamman's sign” [2]. Hamman's sign is not a universal finding and is best heard with the patient in the left lateral decubitus position. Hamman's syndrome is a benign and self-limiting condition that usually affects young males (13–35 years) and pregnant females [3].

Hamman's syndrome may occur after prolonged labor, forceful coughing from asthmatic bronchospasm or pulmonary infections, retching in vomiting, and aggressive physical activity [4]. It has also been associated with inhalational drug use.

Hamman's syndrome is a rare complication of labor and delivery. It has been reported in all stages of labor but usually occurs in the second stage and often is clinically apparent only in the postpartum phase [5]. It has also been seen in the prenatal phase, related to hyperemesis or self-induced vomiting [6]. Previous reports have shown that nulliparity and excessive labor have been associated with this condition.

## 2. Case Report

**Figure 1:**
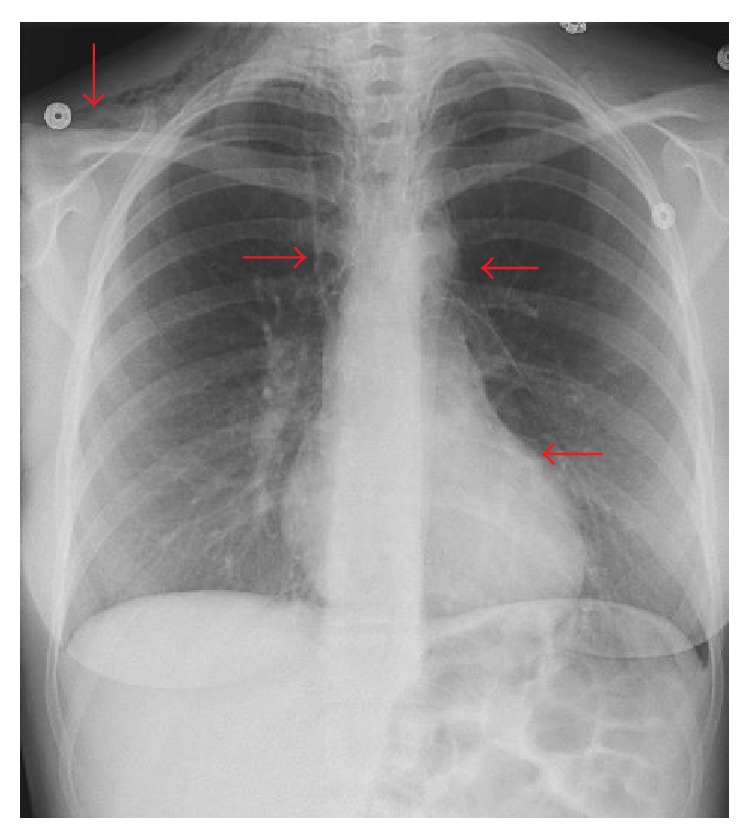
The chest X-ray demonstrates extensive subcutaneous emphysema and pneumopericardium (red arrows).

**Figure 2:**
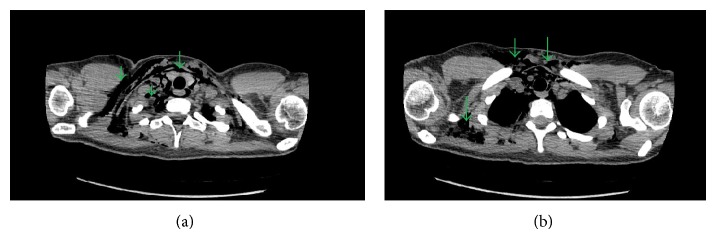
These images are obtained from CT thorax without contrast (mediastinal window) which confirms the findings in the chest X-ray (Green arrows).

**Figure 3:**
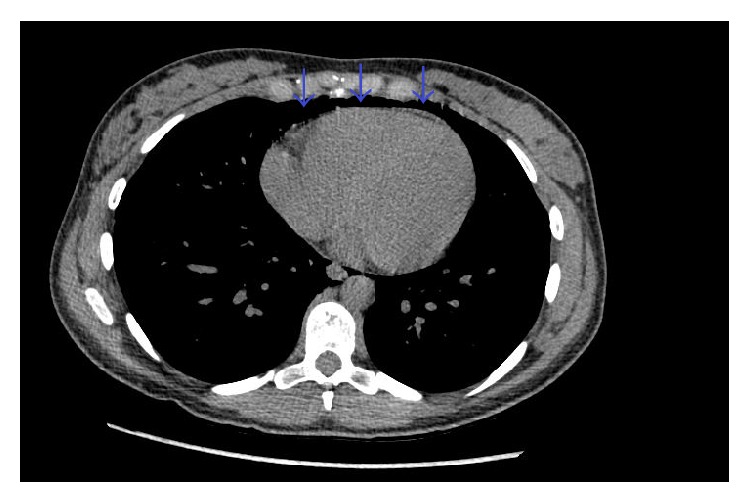
There is an evidence of pneumopericardium without evidence of right ventricular collapse (blue arrows).

**Figure 4:**
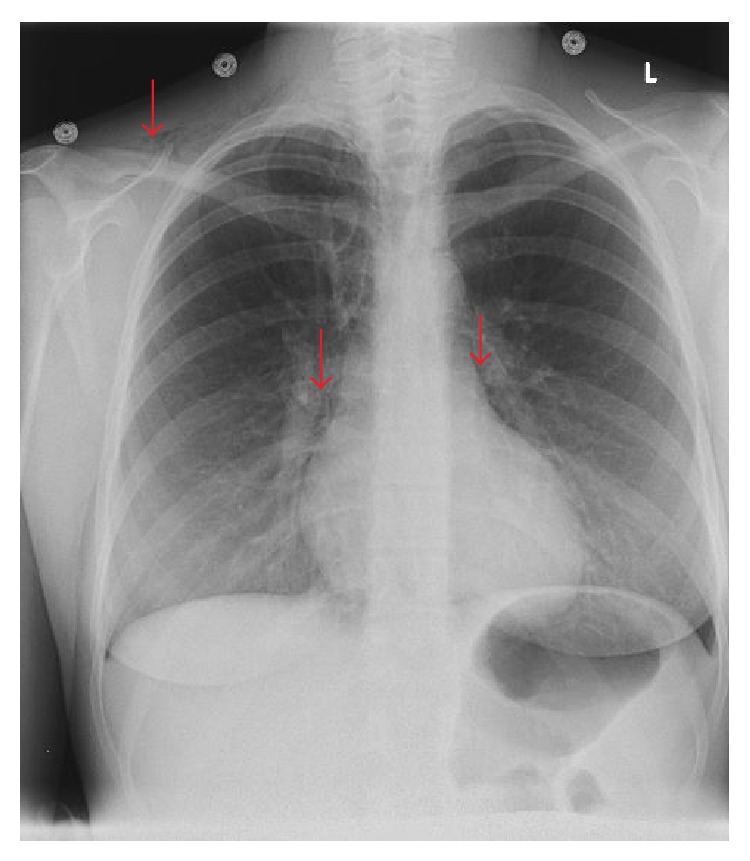
Just prior to discharge, a chest X-ray shows marked improvement in subcutaneous emphysema (red arrows).

A twenty-four-year-old female, gravida 1, para 1, presented to the hospital for induction of labor at 40 weeks and 3 days. Her past medical history was significant for depression. There was no known history of any cardiac condition, throughout her prenatal evaluation. She was taking only prenatal vitamins. She denied smoking cigarettes, drinking alcohol, or using illicit drug. Labor was induced, and she eventually entered the second stage of labor, which was prolonged requiring vacuum-assistance for delivery of the baby. Two hours after the delivery, the patient started to feel neck tightness and right chest tenderness with palpation. She denied any shortness of breath or palpitation. She had normal vital signs and did not require supplemental oxygen to maintain normal O2 saturation. On physical examination, crepitus was felt underneath the skin of the right side of the chest and the neck bilaterally. However, crunching sound was not audible with the heartbeat. Chest X-ray showed pneumomediastinum extending into the right shoulder (Figure 1). There was a concern for pneumopericardium. The CT scan of thorax without contrast revealed extensive subcutaneous emphysema within the right shoulder region extending to the neck (Figures 2(a) and 2(b)) and pericardium (Figure 3). An EKG was unremarkable. A 2D echocardiogram was unrevealing due to interference of air in pericardium; however, it did not show evidence of tamponade. Hamman's syndrome was diagnosed on the basis of the patient's history and clinical findings, supported by imaging studies. As the patient was hemodynamically stable, a conservative approach for management was taken. The patient was observed for 48 hours and discharged home after she remained stable and the follow-up chest X-ray showed decrease in subcutaneous emphysema (Figure 4).

## 3. Discussion

The incidence of  Hamman's syndrome after labor is estimated to be about 1 in 100,000 vaginal deliveries and 1 in 2000 vaginal deliveries [7]. In the last century, there have been around 200 reported cases of Hamman's syndrome during labor.

Spontaneous mediastinum is believed to be a result of the spontaneous rupture of an alveolus and may also be called “respiratory pneumomediastinum” [3]. The pathophysiology was explained by M. T. Macklin and C. C. Macklin in 1944 [8]. Barotrauma causes alveolar rupture and the air tracks along the bronchovascular connective tissue planes into the mediastinum and hilum [9]. This is also called the “Macklin Effect.” Mediastinal air escapes into the subcutaneous tissue, resulting in subcutaneous emphysema. It may extend into the neck and give a feeling of neck tightness, as in our patient.

In the case of labor induced Hamman's syndrome, the prolonged Valsalva maneuver (forced expiration against a closed glottis) during the second stage of labor results in the increase in intra-alveolar pressure and rupture of a marginally situated alveolus into the interstitial space. Coughing, vomiting, screaming, and the force of pushing in labor, all, can increase the intrathoracic pressure. A pressure of up to 50 cm of water has been recorded, which may result in this condition [10].

The most common presenting complaint is retrosternal chest pain, radiating to the back or neck. Dysphagia, dysphonia, and dyspnea may be present [3]. Subcutaneous emphysema is the most common sign [11]. A precordial crunching sound, synchronous with heart beat (Hamman's sign), may also be present, and loss of cardiac dullness will be present on percussion. Hamman's sign was absent in our patient.

Hamman's syndrome is usually benign and nonrecurrent [12]. In rare situations, it may be life threatening and lead to cardiac tamponade. However, other potentially serious conditions need to be ruled out before making the diagnosis. The important differentials include esophageal rupture, pulmonary embolism, amniotic fluid embolism, aortic dissection, and myocardial infarction [13]. In the setting of severe vomiting, esophageal rupture must be ruled out, as they are commonly precipitated by the same factors. This can be done with esophagogram and, in some cases, diagnostic endoscopy [3].

A definitive diagnosis can be made with radiology [14]. Chest X-ray is the initial diagnostic modality. A CT scan provides more accurate information about extension of subcutaneous emphysema. CT is considered the gold standard for detecting mediastinal air, as it can detect small amounts which cannot be seen on a chest X-ray [15].

Once other conditions have been ruled out and Hamman's syndrome is diagnosed, reassurance and supportive measures with oxygen, sedatives, and analgesics are usually sufficient for treatment. In some cases with pneumothorax, a chest tube might be placed [16]. Recurrence is uncommon and responds to conservative treatment [17]. Patients can be safely discharged home if they are well and do not have a significant pneumothorax. Routine follow-up is not necessary [15].

On reviewing the literature, Hamman's syndrome occurs in obstetric females, and in nonobstetric cases, 70.2% of the affected cases are males in their early twenties [18]. It is rare and it predominantly occurs in nulliparous women, although the pathophysiology behind this is unclear. Recently reported obstetric cases have been nulliparous women with either a prolonged second stage of labor or excessive crying during delivery [13, 19]. In males, ethanol intoxication has been considered the significant predisposing factor [15]. Regarding further pregnancies, there are no standard guidelines [19]. However, expectant management and using epidural anesthesia to minimize bearing down by the mother, with instrumentation as per case, have been advised [20].

## 4. Conclusion

Spontaneous pneumomediastinum with subcutaneous emphysema (Hamman's syndrome) is a relatively benign condition and is treated conservatively. It can present in all 3 stages of labor and it is due to increased intra-alveolar pressure resulting in alveolar rupture. In certain cases, it can cause significant hemodynamic compromise from tamponade.
